# Reforms: a quest for efficiency or an opportunity for vested interests’? a case study of pharmaceutical policy reforms in Tanzania

**DOI:** 10.1186/1471-2458-13-651

**Published:** 2013-07-13

**Authors:** Amani Thomas Mori, Eliangiringa Amos Kaale, Peter Risha

**Affiliations:** 1 School of Pharmacy, Muhimbili University of Health and Allied Sciences, P.O. Box 65013, Dar es Salaam, Tanzania; 2 Department of Global Public Health and Primary Care, University of Bergen, P.O. Box 7804, 5020, Bergen, Norway

**Keywords:** Tanzania, Pharmaceutical policy, Politics, Policy reforms, Policy actors, Pharmacy Act, Pharmacy Council, Tanzania Food and Drugs Authority

## Abstract

**Background:**

Regulation of the pharmaceutical sector is a challenging task for most governments in the developing countries. In Tanzania, this task falls under the Food and Drugs Authority and the Pharmacy Council. In 2010, the Pharmacy Council spearheaded policy reforms in the pharmaceutical sector aimed at taking over the control of the regulation of the business of pharmacy from the Tanzania Food and Drugs Authority. This study provides a critical analysis of these reforms.

**Methods:**

The study employed a qualitative case-study design. Data was collected through in-depth interviews, focus group discussions and document reviews. Data was analyzed thematically using a policy triangle framework. The analysis was done manually.

**Results:**

The reforms adopted an incremental model of public policy-making and the process was characterized by lobbying for political support, negotiations and bargaining between the interest groups. These negotiations were largely centred on vested interests and not on the impact of the reforms on the efficiency of pharmaceutical regulations in the country. Stakeholders from the micro and meso levels were minimally involved in the policy reforms.

**Conclusion:**

Recent pharmaceutical regulation reforms in Tanzania were overshadowed by vested interests, displacing a critical analysis of optimal policy options that have the potential to increase efficiency in the regulation of the business of pharmacy. Politics influenced decision-making at different levels of the reform process.

## Background

Policy reform is a complex process which has been described in the literature as being “profoundly political”. Politics are inescapable because reforms affect vested interests and usually tend to redistribute valued benefits and harms among competing groups in a society [1]. Since these competing groups are not equally powerful to influence decision-making [2], it is thus advisable to undertake a stakeholder analysis in order to gain a clear understanding of those who support or oppose the reforms in order to manage the politics of policy reforms [1,3]. Reich (2002), argues that the process of policy change should be approached through politics by applying adequate political skills and proper management of the political process [4].

It is, however, unfortunate that more often than not policy-makers concentrate on the content of the reforms without placing enough emphasis on the actors and politics of the reform process itself [5]. As a result, policy reforms can become problematic and inefficient; such a situation is reflected in the recent reforms of pharmaceutical regulation in Tanzania. Despite being among the most important components of the healthcare system, pharmaceutical reforms in developing countries are amongst the least studied of health reforms [6]. Hence it is not surprising that there is a paucity of literature reviews about policy reforms of pharmaceutical regulations in developing countries.

### Historical background of pharmaceutical regulation in Tanzania

After independence, the pharmaceutical sector in Tanzania was regulated by the Pharmacy and Poisons Ordinance, which came under the Pharmacy Board. The ordinance had undergone several amendments, the last one being that of 1978 [7]. During the early 2000s, the Ministry of Health, initiated structural reforms aiming to improve the regulation of the pharmaceutical sector by separating professional practice from pharmaceutical products. These initiatives established two institutions; the Pharmacy Council and the Tanzania Food and Drugs Authority (TFDA). The establishment of the TFDA and the Pharmacy Council marked a new era in the regulation of the pharmaceutical sector in Tanzania.

### The composition and functions of the Pharmacy Council

The Pharmacy Council was established under the Pharmacy Act No. 7 of 2002 to deal with professional matters. The Act gives the Minister for Health and Social Welfare the power to appoint a pharmacist from the public service to be the Registrar of the Pharmacy Council [8]. The council also has a deputy registrar and a secretariat. The core functions of the Pharmacy Council include keeping the registers of pharmaceutical personnel, monitoring and regulating pharmacy practice and approving training institutions and their curricula [8].

### The composition and functions of the Tanzania Food and Drugs Authority

The Tanzania Food and Drugs Authority (TFDA) was established in 2003, under the Tanzania Food, Drugs and Cosmetics Act No.1 of 2003, with the aim of increasing efficiency in the regulation and control of food, drugs, medical devices, cosmetics, herbal drugs and poisons [9]. The Act gives the Minister for Health and Social Welfare the power to appoint a Director General from among those persons who possess relevant qualifications, experience and expertise sufficient to manage the affairs of the authority. The Act mandated the TFDA to regulate the safety, quality and efficacy of pharmaceuticals under its portfolio. Other key functions include the registration of all premises that manufacture, store and dispense pharmaceuticals [9].

The structural reforms shifted over to the TFDA all the functions of the Pharmacy Board, including its employees and infrastructure. The TFDA also inherited well-defined sources of finance, such as fees charged for registration, import and export of regulated products and a share from Government budget subventions. On the other hand, the Pharmacy Council was a very new institution and started with the appointment of a Registrar as its first employee. With a very limited budget from the Government, the Pharmacy Council found innovative new sources of finance, such as registration and professional fees payable by pharmaceutical personnel and owners of pharmacy premises, respectively.

### Regulatory reforms with the Pharmacy Bill 2010

In April 2010 the Minister for Health and Social Welfare, under the advice and initiatives from the Pharmacy Council, tabled the Pharmacy Bill 2010 to the 9^th^ Parliamentary Session to reform pharmaceutical regulations in Tanzania [10]. The proposed law intended to make amendments to the TFDA Act No. 1 of 2003, in order to transfer the registration and regulation of all premises that manufacture, store and dispense pharmaceuticals from the TFDA to the Pharmacy Council portfolio. This was despite the latter’s limited capacity and experience in regulatory affairs. The bill was withdrawn from Parliament during the later stages after its submission.

In March 2011, the Minister re-tabled the Pharmacy Bill Supplement to the 10^th^ Parliamentary session [11]. After intense debate, the bill supplement was enacted by Parliament as the Pharmacy Act, 2011 [12]. This study analyzes these pharmaceutical regulation reforms in Tanzania by examining how politics, vested interests, power and institutions influenced decision-making at different levels of the reform process.

## Methods

### Study design

The study employed a qualitative case study design, which is an empirical inquiry that investigates a contemporary phenomenon in its real-life context [13]. This design was chosen because policy reforms are complex and specific to social, economic and political conditions [14]. The study adheres to RATS guidelines on qualitative research [15].

### Sampling and study participants

The study employed a purposive sampling method since the aim was to explore the views and perspectives of a broad range of stakeholders who participated in the reforms and those who were likely to be affected by the proposed reforms. Study participants represented training institutions, public and private hospitals, vertical programmes, the Ministry of Health and Social Welfare, the Tanzania Food and Drugs Authority, the Pharmacy Council, the Medical Stores Department, non-governmental organizations and community pharmacists.

### Data collection methods

Data was collected through in-depth interviews with key informants, focus group discussions and document reviews from April 2011-August 2012. Key documents that were reviewed include: the Pharmacy Act 2002, the Pharmacy Bill of 2010 and its supplement, the Pharmacy Act 2011, the TFDA Act 2003, Parliamentary contributions on the Pharmacy Bill 2010, published reports and 60 transcripts that were solicited from *mfamasiatz* – an online discussion forum for pharmacists. In-depth interviews were also held with ten key informants using a pre-tested interview guide to supplement the contributions gathered from the forum under the title “Pharmacy Bill 2010”. The number of key informants was determined by the level of data saturation. Three focus group discussions were held with stakeholders from meso and micro levels to supplement the data collected by document reviews and interviews. Informal conversations were also held *ad hoc* with a number of stakeholders in various places including meetings, conferences, offices and eating places.

### Data management and analysis

Interview data were transcribed and the printed hard copies were kept by the investigator. The analysis was done manually using a thematic approach. Transcripts were read repeatedly to identify conceptually similar fragments of text, which were then analyzed according to the policy triangle framework [5]. Analysis involved triangulation of data collected by various methods.

### Ethical clearance

Ethical clearance was granted by the MUHAS Ethical Review Board. Key informants were asked for consent before being interviewed. Informants agreed to participate in the study on condition of anonymity.

## Results

This section provides the results of the analysis of the pharmaceutical regulation reforms with supporting quotes from the study participants and document reviews. Some informants were given code numbers to maintain confidentiality.

### Agenda setting

Some informants said that the agenda for the reforms had been in the making for a couple of years. They said that in 2006, the Pharmacy Council presented a concept paper at a pharmacists’ conference, highlighting some sections of the TFDA Act of 2003 which they believed needed to be harmonized with its functions. The Pharmacy Council and the TFDA unanimously agreed to convene a meeting under the guidance of the then Pharmaceutical Supplies Unit (PSU) to develop a proposal to be submitted to the Minister for Health and Social Welfare. Within a short time, the two institutions managed to identify key sections that they proposed to discuss in the following meetings. Informants said that the Pharmacy Council unilaterally forwarded the proposal they were working on with the TFDA to the Minister of Health and Social Welfare for further action.

*I remember the two institutions were working closely together after that conference on how to harmonize some of the problematic areas of the TFDA Act No*. *1 of 2003*. *There was a meeting and we produced a paper highlighting specific sections which we thought we were supposed to work on*. *The section about premise registrations was one of them*. *I do not know what happened afterwards*, *we came to realize that the paper was already at the Ministry of Health and Social Welfare*, *we were all stunned*! (Participant 3)

### The content of the Bill and the enacted Pharmacy Act, 2011

The proposed bill aimed to repeal section 21 of the TFDA Act No. 1 of 2003, which deals with the registration of premises and issuance of permits for businesses dealing with medicines [9]. The bill proposed that the Pharmacy Council should have the mandate to register premises and issue permits for the sale, dispensing and supply of medicinal products. These permits were categorized as retail, wholesale, institutional or of any other business as the Council may deem necessary for the purposes of the Act [10]. We learned through the interviews and document reviews that behind this “goodwill” to improve the regulation of the business of pharmacy there was a hidden interest by pharmacists. Many pharmacists who contributed about the topic in the discussion forum shared the same view that the proposed law should be supported because it protects the interests of pharmacists. One of them commented:

*One of the core functions of the Pharmacy Council is to protect the interests of pharmaceutical personnel*, *and since the Pharmacy Council will always have a registrar who is a pharmacist then we are safe*. *So why not put the premises under the Pharmacy Council to secure our interests from other professions*? (Participant 15)

Document reviews showed that, since the enactment of the TFDA Act No. 1 of 2003 and its subsequent implementation, the Government of Tanzania, through the Ministry of Health and Social Welfare, has invested a lot of resources in building up the capacity of the TFDA as a regulatory authority to protect its people from sub-standard and counterfeit medicines. This includes a significant increase in the number of employees, from 52 in 2003 to almost 200 by 2011, to run the new operations provided in the portfolio.

In 2006, the Ministry of Health and Social Welfare delegated to the Prime Minister’s Office, Regional and Local Government (PMO-RALG), some of the powers and functions of the TFDA through the Tanzania Food & Drugs Delegation of Powers Order of 2006, amended in 2007 [16]. The TFDA had worked with PMO-RALG to establish Council Food and Drug Committees (CFDC) that were mandated to discharge some of the regulatory functions related to operating premises (other than pharmacies) dealing with the sale of pharmaceuticals in their locality. In the long term, this would have decentralized regulation closer to where it was needed, since the majority of the Councils had already established CFDCs with a reporting structure to the TFDA.

To a large extent, those who were against the policy reforms were cautious about the capacity of the Pharmacy Council to deal with the regulation of pharmacy businesses. Many felt there was no justification for the Pharmacy Council to engage with the business of pharmacy since the TFDA was already making good progress in that area. One Member of Parliament warned that these reforms would have counterproductive consequences, hence compromising the achievements of the TFDA in that area [17].

*All of Section IV of the bill which deals with the business of pharmacy should be removed*; *the bill should deal with professional issues only*. *This is how we can do justice to our nation*; *otherwise we will enter into major problems*. *We want to start new things for no good reasons*, *the Ministry of Health and Social Welfare must tell us what were the shortcomings observed when the business of Pharmacy was under the TFDA*, *that*’*s the only way we can endorse this bill*. – Parliamentary Contribution to the Pharmacy Bill 2010 [18].

In spite of strong opposition from Members of Parliament, the enacted Pharmacy Act, 2011, retained Section IV of the bill, which authorizes the Pharmacy Council to register premises and issue permits for businesses dealing with medicines. Specifically, the Pharmacy Council was mandated to issue permits for retail, distribution, institutional or any other business as it may deem fit for the purposes of the Act, while the TFDA was left with the manufacturing and wholesale permits [12]. In our opinion, this decision was reached to create a win-win situation for the two institutions but unfortunately created more contradictions among their clients. While the main responsibility of the Pharmacy Council is to regulate professional practice and training, some stakeholders wondered how this function relates to the registration of premises. It is also not well understood how the once contradictory bill was passed into an Act despite strong opposition from Members of Parliament to cede the TFDA’s roles to the Pharmacy Council [17].

### Process

The process that produced the bill and its subsequent submission to Parliament was not transparent and was conducted *ad hoc*. The process was initiated by the Pharmacy Council as an agenda setter. The proposed bill was endorsed by the Ministry of Health and Social Welfare, which submitted it to Parliament. The bill was returned to the Parliamentary Standing Committee for Social Welfare (PSCSW) for more discussions with stakeholders, who were perceived not to have been involved to a sufficient extent. Some of the informants who participated in the discussions about the bill within the PSCSW said that they witnessed a lot of lobbying activities for political support among the two rival groups.

*It was all about lobbying for political support*, *some people lobbied to be part of the committee*, *others lobbied the members of the committee from outside*, *and it was chaotic*! (Participant who attended PSCSW meeting)

### Stakeholder involvement

There were both visible and hidden participants in the policy review process; hence, it was difficult to know exactly who was involved and who was not. However, officials in the Pharmacy Council, the TFDA and the Ministry of Health and Social Welfare were the most prominent actors behind the pharmaceutical regulation reforms. The Pharmacy Council as the agenda setter, along with the Ministry of Health and Social Welfare, supported the reforms while the TFDA opposed them. The TFDA was accused of working through informal political channels to block the bill and some officials were even threatened with dismissal from their positions.

Stakeholders at the micro level, such as community pharmacists and owners of premises, were minimally involved. Informants said that only a few representatives, particularly from the pharmacy training institutions, were involved. In an attempt to address concerns about the inadequate involvement of stakeholders, the Parliamentary Standing Committee for Social Welfare invited some stakeholders to discuss the content of the bill after it had been removed from Parliament. Information about who was invited was not made public and it was not obvious whose opinions these representatives were going to convey in the meeting. It was during this time that there was an outcry from the community of pharmacists that the bill was again being treated with secrecy without their full involvement. The bill was not made available to the public and it was only through informal channels that hard copies were accessible. The opinions of stakeholders who were not invited by the Parliamentary Standing Committee for Social Welfare were unlikely to be chanelled onto the discussion table.

(…) *of all the things*, *the most interesting and annoying one is where stakeholders were bypassed and instead lobbyists went to Parliament in Dodoma*. *Is this where we have reached*! *What were they worried about*? *Everything with this bill suggests it is for personal interests and short sightedness*. (Participant 50)

## Discussion

This study shows that, as has previously been documented, policy reform is largely a political process and that politics affect the origins, formulation and implementation of public policy [1]. The first bill was withdrawn from Parliament after its submission because the opposing group was powerful enough to block it from going through. This scenario has been explained in the literature in terms of actors having unequal power to influence decision-making [2] and that some are better placed in the system than others to block proposed reforms [5]. The influence of politics twisted the flow of events and dramatically sculpted the reforms to match the interests of the most powerful stakeholders [14]. These vested interests and powers ensured that a compromise deal was struck whereby the Pharmacy Council took over the regulation of retail premises while the TFDA retained control of wholesale and manufacturing premises [12].

It is unfortunate that the Pharmacy Council as the agenda setter did not recognize the influence of political power in the reform process. In managing the politics of competing groups in policy reform, a clear understanding of who is supporting the reform, who is against it, and who is not mobilized in one direction or the other is a critical point that can never be overlooked [1]. With clear information on power differences, policy reforms must involve political negotiations between the key actors affected by the reforms. Negotiations must proceed with great caution to avoid ending up with a policy reform proposal that reflects the interests of the key actors at the expense of more feasible and efficient solutions.

This study reveals a typical example of the incremental model of policy-making. In this model, bargaining, negotiations and adjustments between interest groups play major roles in determining decision outcomes, displacing a critical analysis of optimal policy options [19]. Decision-making in the incremental model is to large extent a political activity rather than a technical one and the outcomes differs only marginally from the existing ones [19,20].

The study also shows that the interests and preferences of a small group of elite actors influenced decision-making [2]. The Pharmacy Council claimed to have invited representatives from various institutions and groups to convey ideas at the policy table; however, as was reported in another study, the said representation was neither adequate nor sufficient [21]. Prior to the submission of the proposed bill to Parliament, there was supposed to be extensive and comprehensive stakeholder discussion to build consensus. Because of the lack of consensus among the competing groups, the reform process was difficult, time-consuming and used more resources than had initially been predicted. Use of other methods, such as professional association meetings and interviews with stakeholders, could have been used to gather information. This would have helped to shape the content of the reform before tabling it in Parliament.

### Strengths and limitations

One of the major strengths of this study is that data were collected by different methods and were triangulated during analysis to increase the validity of its findings. Despite this, the study had two major limitations; firstly, qualitative methods are prone to information bias, which can only be minimized but not eliminated. For example, during interviews informants may forget valuable information which is relevant to the study. Secondly, we might have missed to interview some people whom we believe may possess important information related to this study, although we are optimistic that their opinions would not have changed our conclusion.

## Conclusion

Recent pharmaceutical regulation reforms in Tanzania were overshadowed by vested interests displacing a critical analysis of optimal policy options that have the potential to increase efficiency in the regulation of the business of pharmacy. Politics influenced decision-making at different levels of the reform process. As such, regulation of the business of pharmacy remains a major challenge in Tanzania. The government should intervene by putting in place a better regulatory mechanism aiming to protect the public interest. The government should also be more vigilant about limiting the influence of political power in policy reform processes to avoid the compromise of efficiency by personal interests. Otherwise the risks of undoing previous strides made in pharmaceutical regulation are very great and real, and this would have a devastating impact on public health.

## Competing interest

The authors have received no financial or non-financial considerations in relation to the work reported in the manuscript. EAK is a member of a technical committee for the registration of human medicine for the TFDA. Both authors are registered pharmacists by the Pharmacy Council.

## Authors’ contributions

ATM designed the study, participated in data collection, carried out the data analysis and drafted the manuscript. EAK carried out the interviews, supervised data analysis and helped to draft the manuscript. PGR participated in the design of the study, supervised the overall implementation and helped to draft the manuscript. All authors read and approved the final manuscript.

## Pre-publication history

The pre-publication history for this paper can be accessed here:

http://www.biomedcentral.com/1471-2458/13/651/prepub
